# Fin Whale (*Balaenoptera physalus*) Diel Dive Patterns in a Shallow Fragmented Foraging Habitat

**DOI:** 10.1002/ece3.74313

**Published:** 2026-09-10

**Authors:** Dana L. Adcock, Douglas P. Nowacek, Susan E. Parks

**Affiliations:** ^1^ Department of Biology Syracuse University Syracuse New York USA; ^2^ Nicholas School of the Environment & Pratt School of Engineering, Duke University Marine Lab Beaufort North Carolina USA

**Keywords:** behavioural ecology, conservation, fin whale, foraging ecology, habitat fragmentation, traffic

## Abstract

Predators select foraging habitats to maximise energetic gains. However, habitat fragmentation can lead to increased costs from disturbances in high traffic urban environments. Understanding behavioural drivers of movement ecology is crucial for minimising conflict between evolutionarily selected foraging behaviours and novel anthropogenic stressors. In this study, 27 high‐resolution biologging tags were deployed on fin whales in a shallow inner‐shelf habitat south of the US east coast, where water depth can be less than the body length of the animal. The vertical movement patterns of the whales from diving overlapped with major shipping traffic. The whales spent > 67% of their time at depths shallower than the drafts of large ships (~10 m). The whales fed primarily during the day and remained very shallow at night (mean 5 m) while resting and travelling, possibly due to diel changes in vertical prey distribution. This natural behavioural pattern has become maladaptive with the introduction of anthropogenic traffic and puts them at extreme risk of injury and mortality in commercial shipping lanes at night, when mariners have limited visibility to detect them. These results underscore the importance of understanding baseline vertical movement patterns and behavioural trends to inform conservation efforts in fragmented environments.

## Graphical abstract

Using 27 high‐resolution biologging tags deployed on fin whales in a shallow inner‐shelf habitat along the US east coast, this study found that the whales spent more than 67% of their time within the draft depths of large commercial vessels. Feeding occurred primarily during the day, while whales remained very shallow at night during resting and travelling, likely tracking diel shifts in prey distribution. These results suggest that an evolutionarily selected foraging strategy in a productive habitat has become maladaptive in the presence of heavy shipping traffic.

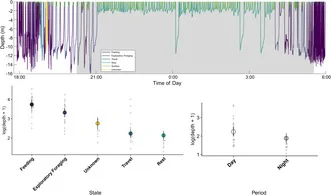

## 1 Introduction

Advantageous foraging behaviour develops from both heritable transgenerational effects and individual experiences (Des Roches et al. 2022). Foraging strategies are predicted to maximise energetic gains and limit costs during the pursuit and acquisition of prey, as defined by the optimal foraging theory (Pyke 1984, 2010). Predators maximise gains by targeting spatial and temporal peaks of high‐value prey (Krebs et al. 1977; Iwasa et al. 1981). Foraging costs are determined primarily by prey abundance and distribution, nutritional richness and handling time (Emlen 1966; Goldbogen et al. 2023). These costs may be higher for predators that invest energy chasing and handling prey, for example, ambush predators (deVries et al. 2012; Hubel et al. 2016; Goldbogen et al. 2023), particularly if there are risks of injury (Brown and Kotler 2004). Evolutionary pressures select for foraging behaviour that maximises intake and minimises costs, resulting in optimal net energetic gain.

While foraging behaviours evolved through natural selection, novel urbanisation pressures may reduce foraging success. Highway noise can reduce a predator's ability to acoustically detect mobile prey (Siemers and Schaub 2010; Passarotto et al. 2025), leading to increased time spent hunting, even if foraging success is not significantly impacted (Ruiz‐Villar et al. 2024). In generalists, higher levels of urbanisation may reduce the diversity of foraging sites and gut microbiota, such as in the seabird *Larus argentatus* (Fuirst et al. 2018). Anthropogenic sounds have been shown to disrupt foraging behaviour in some cetacean species (Tyack et al. 2011; Madsen et al. 2015; Blair et al. 2016; Wisniewska et al. 2018; Miller et al. 2022).

Foraging behaviour can increase mortality in urbanised habitats. Habitat fragmentation breaks up suitable habitat into smaller areas (Yeager et al. 2020), quantified frequently through patch size changes, connectivity loss and increased edge effects, and has been studied in marine systems (Harishchandra et al. 2022; Yeager et al. 2020) and high‐traffic terrestrial systems (Santos et al. 2016; Poulin et al. 2023). Despite the ocean's three‐dimensional environment, changing risk conditions can alter suitable habitat. Habitat fragmentation from traffic in urbanised areas can lead to increased mortality risk while evolutionary responses remain the same (Bennett 2017; Denneboom et al. 2024). Mortality of passerine bird species near roads is strongly related to foraging behaviour (Santos et al. 2016). Woodchucks in urban areas foraged at similar distances from burrows as rural areas, despite high mortality risks from vehicle collisions, demonstrating a lack of habituation and instead tolerance due to the need to forage (Lehrer et al. 2012). In contrast, female elk ( *Cervus canadensis* ) can balance rewards of greater foraging biomass and the risk of crossing high‐volume highways (Poulin et al. 2023). Shallow subsurface feeding of North Atlantic right whales ( *Eubalaena glacialis* ) tracking concentrations of prey in Cape Cod Bay increases vessel collision risk (Parks, Warren, et al. 2011).

Foraging grounds are vital for the survival and successful reproduction of baleen whales, which are capital breeders, in the marine environment. Highly efficient filter feeding in baleen whales (*Mysticeti*) allows for exploitation of body size extremes (Goldbogen et al. 2019). Oxygen storage increases with their body size, allowing them to perform longer dives to engulf prey at depth (Goldbogen and Madsen 2018). Adult baleen whales maximise blubber volume, a measure of energy stores, through enormous energy inputs gathered on the foraging grounds (Brodie 1975; Christiansen et al. 2013). Large energy stores are crucial to support fasting during the breeding season and for thermoregulation (Brodie 1975). As important marine consumers (Jory et al. 2021), the largest adult baleen whales evolved highly productive behavioural activity budgets on foraging grounds with limited predation risk due to their large size (Branch 2025).

However, in the present day, whales are at high risk of injury and mortality from ship collisions, as 92% of their habitat overlaps with maritime traffic (Schoeman et al. 2020; Nisi et al. 2024). The most severe injuries to marine mammals from collisions result from large ships, > 80 m in length (Laist et al. 2001). The waters of Southern New England and the New York Bight are an important shallow coastal habitat (< 100 m maximum depth) on the continental shelf in the Western North Atlantic (Whitehead and Carlson 1988), where fin whales feed on schooling fish during the summer (Staudinger et al. 2020) but are detected acoustically year‐round (Estabrook et al. 2025). Risk for injuries from shipping in this region are driven by the high levels of coastal traffic associated with the port of New York & New Jersey, which consistently ranks as the largest U.S. trading port on the US east coast (Thorne and Wiley 2024; Port Authority of New York and New Jersey, n.d.; International Transport Forum 2019). The drafts of cargo vessels occupy more of the water column space (draft ca. 10 m) than smaller vessels, which raises concern for risk to protected species (Barkaszi et al. 2021; Indeck et al. 2025; Garrison et al. 2022). Ship collision studies largely focus on two‐dimensional or course data for modelling (Aschettino et al. 2020; Nisi et al. 2024). Few studies (Parks, Warren, et al. 2011; Calambokidis et al. 2019; Keen et al. 2019) have examined how three‐dimensional behaviour increases human–wildlife conflict with large baleen whales, particularly in shallow foraging habitats.

This study examines the behavioural drivers of dive depth in fin whales ( *Balaenoptera physalus* ) during their summer feeding season in Southern New England and the New York Bight, a shallow foraging habitat with dense maritime traffic, where vertical space for safe manoeuvrability is limited when co‐located with vessels. We hypothesise that foraging states will dictate overall and diel trends in water column usage. The results will provide a better understanding of evolutionary advantageous three‐dimensional foraging behaviour to inform mortality risk modelling of biologically important areas, particularly for threatened species.

## 2 Methods

### 2.1 Data Collection

**FIGURE 1:**
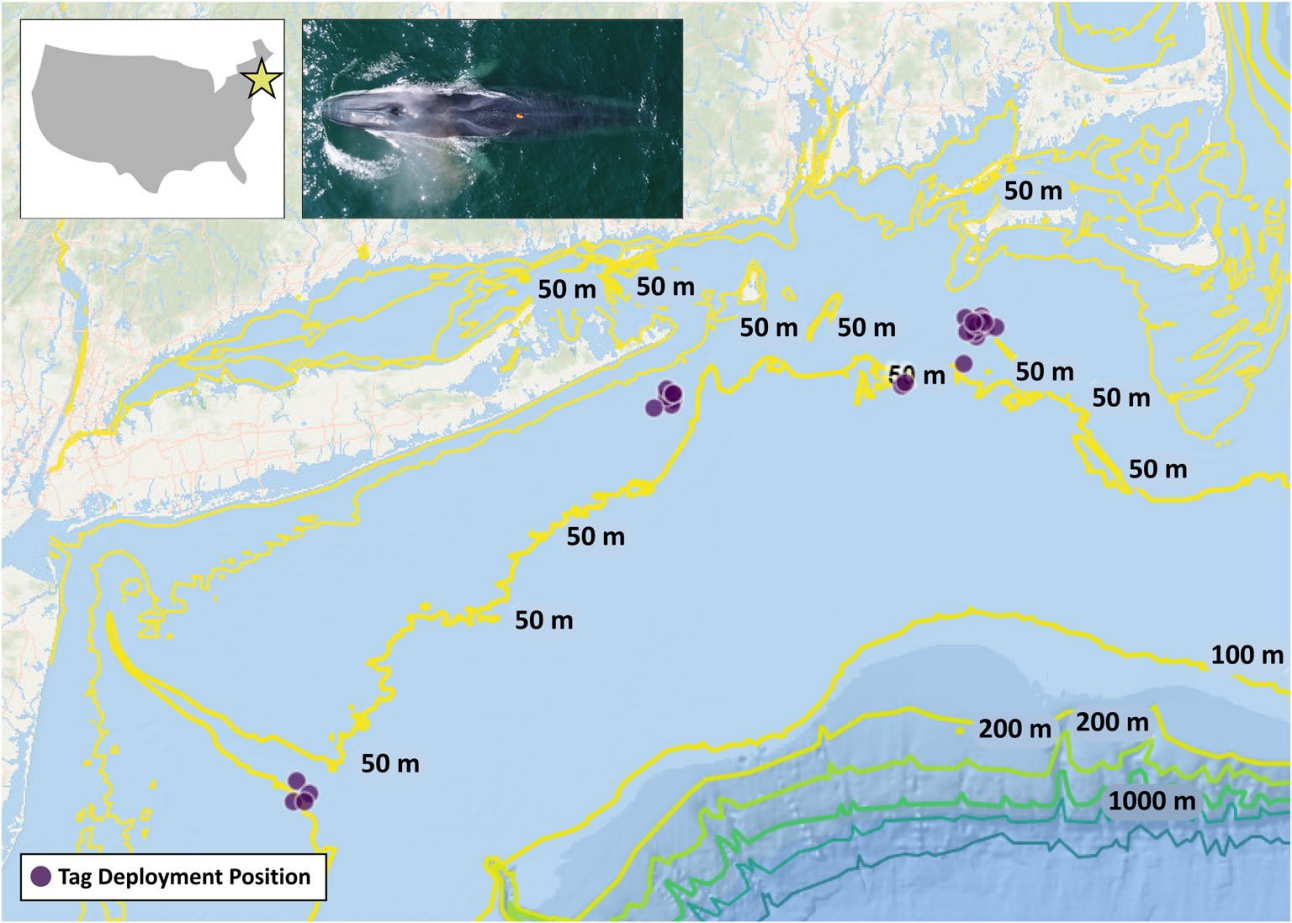
Study area map. Bathymetry contours in New York Bight and Southern New England waters displaying shallow depths in the area from the NOAA NCEI GEBCO Contours dataset (GEBCO Compilation Group 2025). Tag deployment locations are plotted as points. Figure includes a photo example of a tagged fin whale from this study. Photographer: Chris Zadra, Ocean Alliance under NMFS Permit #27272‐01. Base map ‘Ocean’ is the intellectual property of Esri and is used herein under licence. Copyright 2026 Esri and its licensors. All rights reserved.

North Atlantic fin whales were tagged south of Massachusetts and offshore of New York (39.83 to 41.11 latitude, −73.15 to −70.74 longitude) between July 22 and August 3 of 2023 and 2024 from the 21 m research vessel *R/V Song of the Whale* (Figure 1). Fin whale tagging and biological samples were collected under the National Marine Fisheries Service Permits 27272‐01, 23644‐03 and 22156‐04 and IACUC approval. Whales were located through visual surveys and tagged with digital acoustic recording tags (DTAGs) and Customised Animal Tracking Solutions (CATS) tags (Johnson and Tyack 2003; Goldbogen et al. 2017) that were deployed via an unmanned aerial vehicle (UAV) (Wiley et al. 2023) and attached with sterilised suction cups. The DTAGs recorded sound (sampled at 64 kHz, 120 kHz and 192 kHz with frequency ranges of 0–32 kHz, 0–60 kHz and 0–96 kHz), depth (sampled at 50 Hz) and body orientation (sampled at 250 Hz for version 3 and 500 Hz for version 4) and GPS data. GPS locations were obtained from a snapshot GPS receiver during surfacings, ARGOS satellite GPS positions, and the internal GPS from DTAG v4, depending on the tag type and deployment circumstances. GPS positions were obtained for all tags; however, the number of points available varied by deployment due to tag position on the whale affecting how long the tag was above the water's surface. The CATS tags recorded acoustic (sampled at 48 kHz), depth (sampled at 50 Hz) and body orientation (sampled at 400 Hz), and video (4 k and 1080p resolution) data. After tags were deployed, focal follows were performed from an A‐frame 5 m above the water's surface for a subset of deployments. Observers recorded behaviour, group composition and nearby vessels for at least one hour after attachment. After tags detached from the whale and were recovered, skin samples were collected from the suction cups for sex identification (Adcock et al. 2026). UAV footage of the central chevron pattern and blaze, which are stable identifying marks for this species, was used to identify tagged individuals (Adcock et al. 2026). Acoustic behaviour of the tagged whales was assessed in a separate study (Adcock et al. 2026).

### 2.2 Trends in Dive Depth

The study took place on the continental shelf and the mean depth in the habitat was 43 m in Southern New England and 49 m in the New York Bight (Figure 1) (GEBCO Compilation Group 2025). The tag files were processed in MATLAB to produce the dive profile, pitch, roll, heading, and jerk or overall dynamic body acceleration (Johnson and Tyack 2003; animaltags.org). Dives were extracted using the find_dives function (animaltags.org) and those less than 30 s in duration were removed. The first two dives were excluded from the analysis to account for any potential reactions to the tagging event (Hooker et al. 2001).

The proportion of time the tag on the back of the fin whales spent at various depths was measured to assess the potential for ship strike risk. The pressure sensor data were used to measure the amount of time each fin whale spent in 5 m bins, from 0 m to 100 m. The proportion of time each fin whale spent in each bin was calculated. These times were summed together to calculate the proportions for all sampled whales.

**FIGURE 2:**
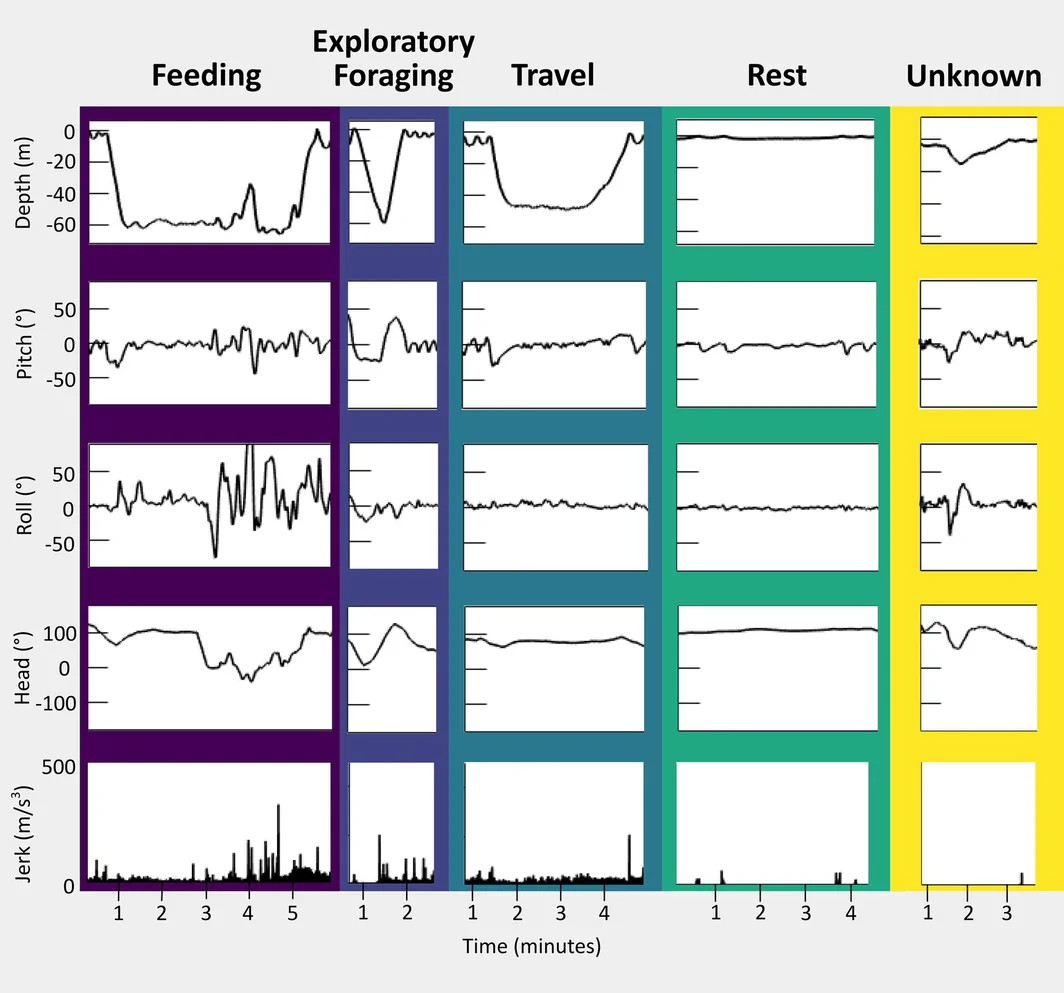
Kinematic examples of behavioural states. Sensor data calibrated to whale frame for classification of five different behavioural states (feeding, exploratory foraging, travel, rest and unknown). Sensor data from top to bottom are as follows: Depth (m), pitch (degrees), roll (degrees), heading (degrees) and jerk. Jerk refers to the rate of change of acceleration over time. All sensor data measurements across time in minutes for about one dive.

Behavioural states (from vertical and horizontal movement and body orientation) were categorised into six categories (feeding, exploratory foraging, travel, rest, surface and unknown) based on focal follows of surface behaviour, underwater CATS video and existing literature (Goldbogen et al. 2006; Parks, Searby, et al. 2011; Friedlaender et al. 2013; Heide‐Jørgensen et al. 2013; Fonseca et al. 2022; Colson et al. 2025). CATS tag data provided information on foraging behaviour, prey type and the presence of other whales. Due to the shallow environment, a dive was defined as a submergence greater than 1 m and longer than 30 s, with this ‘dive’ as the unit of analysis (Colson et al. 2025). Any periods at depths less than 1 m and greater than 30 s (i.e., extended surface intervals) were categorised as surface behaviour. Behavioural states were categorised using kinematic signatures during the dive. Foraging was characterised by accelerometer and jerk signatures for lunge feeding, large changes in pitch, deep dives, spikes in roll for side‐lunges and V‐shaped exploratory dives for food‐finding near lunges (Figure 2) (Goldbogen et al. 2006; Friedlaender et al. 2013; Heide‐Jørgensen et al. 2013; Fonseca et al. 2022). These were split into two states: feeding (lunge present) and exploratory foraging (no lunge present) (Figure 2). Since fin whales accelerate in bursts to reach their prey (Goldbogen et al. 2006), a lunge was defined as the period between a relative velocity maximum and the average velocity. Travelling states had relatively consistent headings, fluke signatures before the dive, and no feeding events (Figure 2). Small amplitude oscillations in the *x*‐axis of the accelerometer correspond to fluke strokes (Goldbogen et al. 2006). In contrast, resting periods were characterised by gradual changes in heading, shallow dives and low activity (Figure 2). Longer periods of this were validated with GPS tracks and overall displacement (Figure S1). Other periods characterised by diverse non‐foraging activity (Parks, Searby, et al. 2011) may represent social behaviour but were marked as unknown (Figure 2).

Mixed models (Gaussian with identity link) were used to test whether the behavioural state impacted maximum dive depth using the lme4 and lmerTest packages in R 4.3.2 (Kuznetsova et al. 2020; Bates et al. 2025). Paired fin whales appeared to display moderate dive synchrony, so pairs where both were tagged were included as a random effect in modelling (Adcock et al. 2026). Maximum dive depth was set as the response variable, behavioural state as a fixed effect and individual and group association as random effects to help account for autocorrelation of data (model selection in Table S1). State levels were feeding, exploratory foraging, travel, rest and unknown. The surface state was excluded as it is correlated with depth (< 1 m). A log(y + 1) transformation was applied to improve the right skewed homogeneity of variance and normality. Using the R package afex, model‐predicted means and confidence intervals of maximum depth were extracted for each predictor to account for pseudoreplication among individuals and groups (Singmann et al. 2024). Therefore, our summary statistics estimates are presented to account for our random effects. These outputs were back‐transformed to reflect original data scales. These means are used to report estimated average maximum dive depth for each fixed predictor.

Using data specific to the tagging locations and dates, diel periods were split into day (time between sunrise and sunset) and night (time between civil twilights), omitting twilight periods between them (https://www.weather.gov/box/sunmoon). Twilight was excluded due to the diel migration of their prey, sand lance, during this period (Baker et al. 2023), which may influence dive behaviour, and there was not sufficient data to analyse this short period alone. The modelling methods above (including transformations) were applied for diel period (levels: day or night) and maximum dive depth.

### 2.3 Behavioural State Diel Trends

The duration of each dive and the time spent in each behavioural state was calculated for all individuals during the day and at night, and then they were transformed into proportions (out of 100% for each diel period). Trends were examined with Pearson's Chi‐Squared Test and pairwise comparisons in R. Associations were calculated using Cramer's V and applied bootstrapping to calculate confidence intervals (Bauer et al. 2015; Rendell and Whitehead 2003).

## 3 Results

### 3.1 Data Collection

**FIGURE 3:**
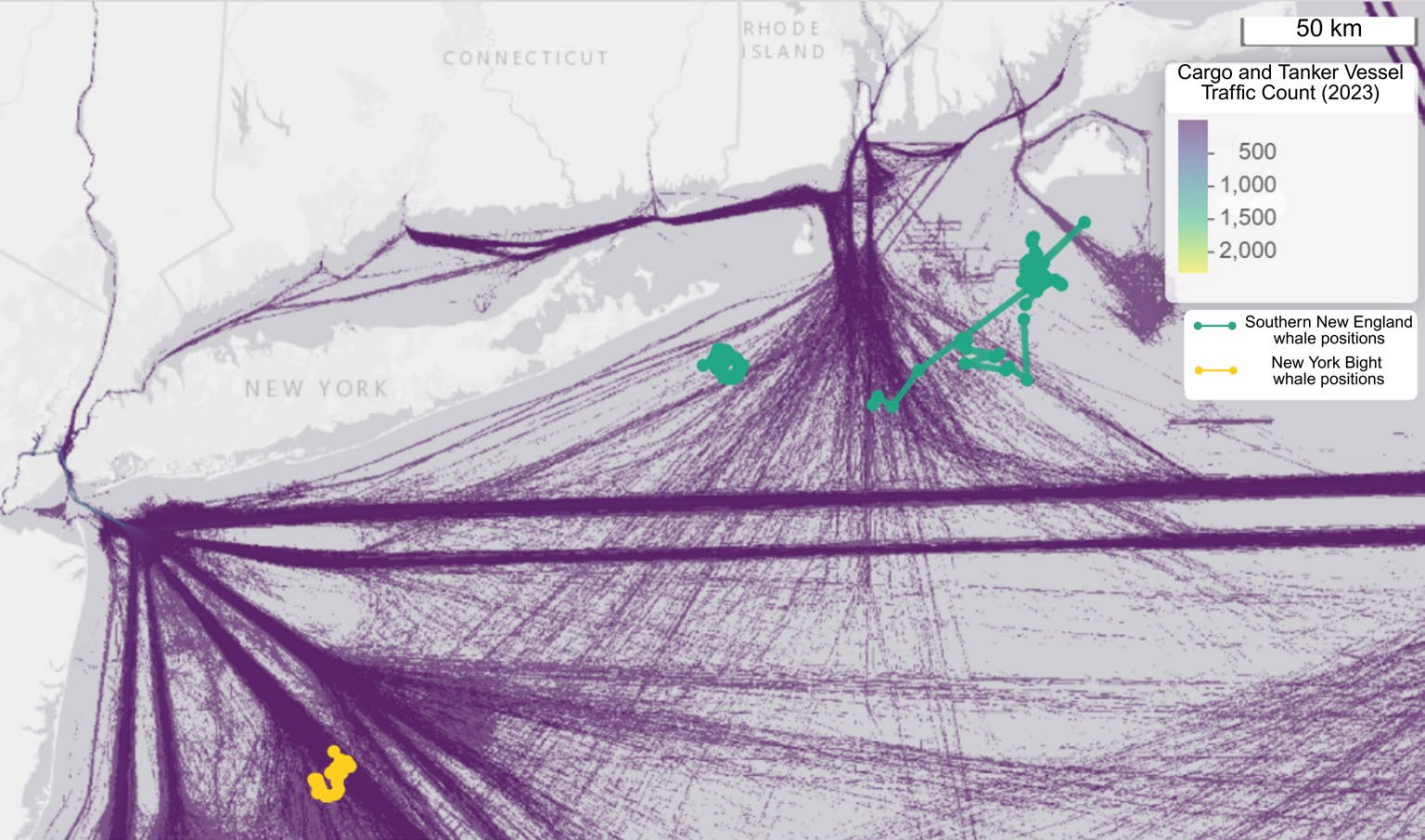
Locations of tagged whales near cargo and tanker traffic. GPS positions and deployment locations for 2023 and 2024 tagged whales (points and lines, southern New England in blue and New York Bight in yellow) mapped alongside 2023 cargo and tanker traffic (density pixels; northeastoceandata.org). Base map ‘Light Gray Canvas Base’ is the intellectual property of Esri and is used herein under licence. Copyright 2026 Esri and its licensors. All rights reserved.

A total of 131 h of acoustic, depth, orientation and GPS data from 27 deployments were collected. Attachment duration mean was 6 h ± 10.2 h with 64.5 h of data recorded during the day and 59.6 h recorded at night. A total of 10.9 h of behavioural visual observations and 19.3 h of CATS tag video were recorded. On video, the whales were observed to feed only on sand lance (*Ammodytes sps*). One individual foraged with a closely associated conspecific from late afternoon into the evening but was alone at dawn when the video resumed. There were 21 unique individuals tagged, 4 of which were tagged two times, and 1 three times across the 2 years. Of these 21 individuals, 20 were male and 1 was female (Adcock et al. 2026). GPS locations overlapped with major shipping lanes offshore of New York and Rhode Island (Figure 3).

### 3.2 Trends in Dive Depth

**FIGURE 4:**
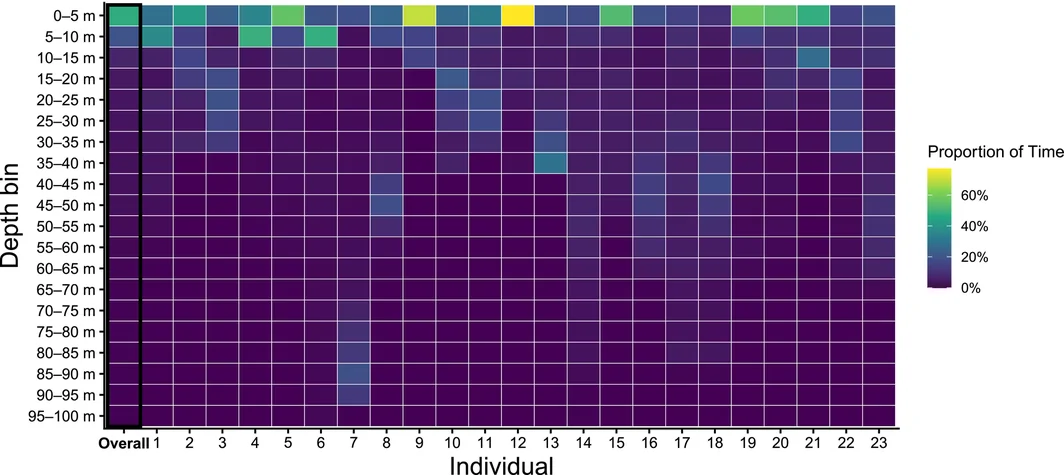
Proportion of time at depth. Proportion of time spent in 5 m depth bins from 0 to 100 m. Lighter yellow represents a larger proportion of time, while darker purple represents a smaller proportion. All time summed together is represented as ‘overall’ and is boxed in black. Most time (74.2%) is spent in less than 15 m of water.

Overall, fin whales spend 47.4% of their time with their backs at less than 5 m depth (Figure 4). A total of 19.8% of time was spent in 5 to 10 m depth; 7.0% is spent in 10 to 15 m depth; 4.2% in 15 to 20 m; 3.5% in 20 to 25 m; 2.8% in 25 to 30 m; 2.8% in 30 to 35 m; 2.6% in 35 to 40 m; 2.5% in 40 to 45 m; 2.3% in 45 to 50 m; 1.4% in 50 to 55 m; and 1% or less in each remaining 5 m bin (Figure 4).

**FIGURE 5:**
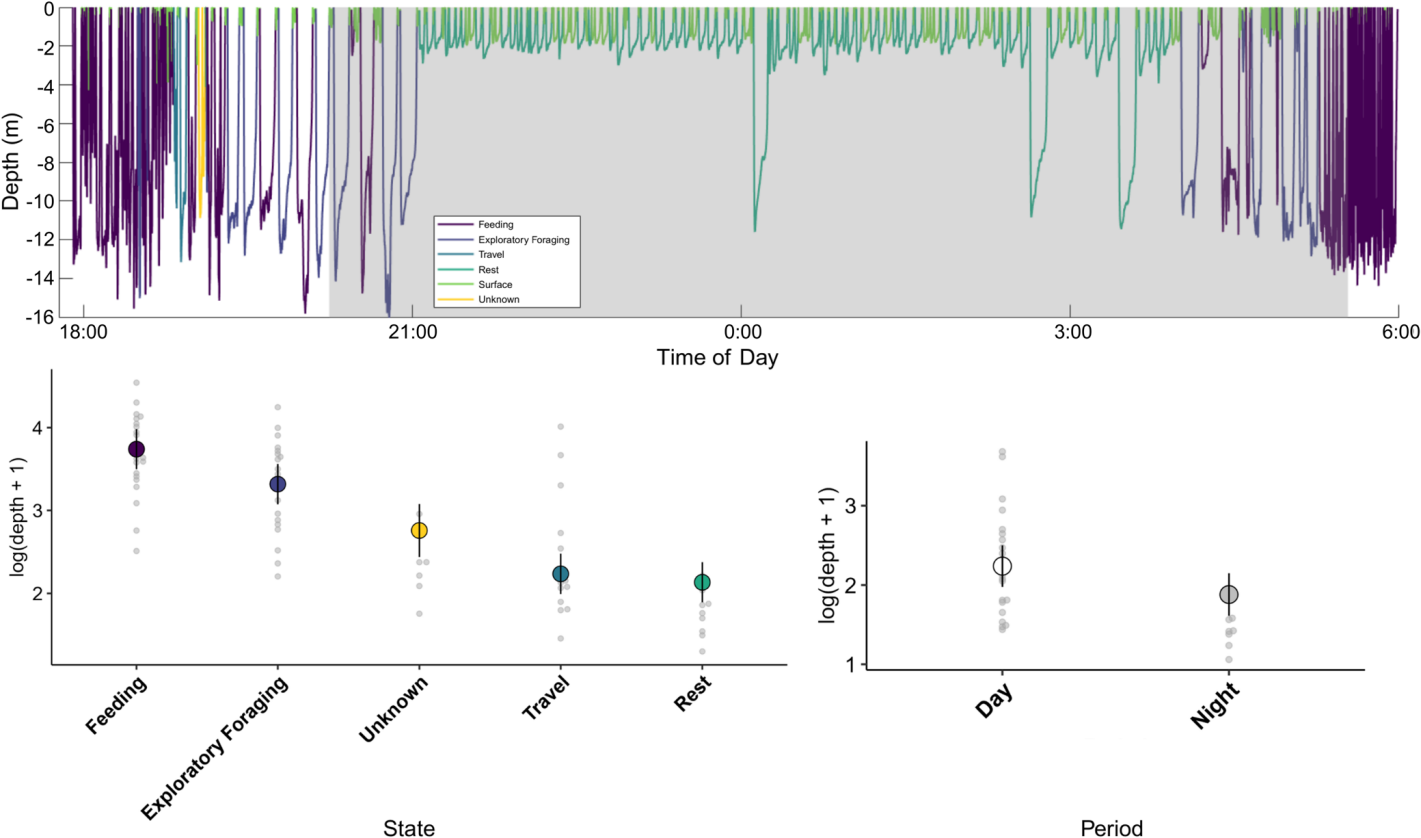
Trends in maximum dive depth. Top panel: Depth over time for an overnight deployment, with night shaded in grey and dives colour‐coded by different behaviours. Bottom left: Model predicted means of maximum dive depth (log(depth) + 1) in each behavioural state. Fin whales have the deepest maximum depth in a feeding state and shallowest in a resting state. Bottom right: Model predicted means of maximum dive depth (log(depth) + 1) during each diel period. They dive deeper during the day than at night. Error bars represent 95% confidence intervals for estimated means.

Diel period was a significant predictor of maximum dive depth in our first linear mixed model and had a small model effect size (*ω* ^2^_*p*_ = 0.02). Both diel periods were significant in the model (df 1, *p* < 0.01), where maximum depth of dive decreases at night (model estimate mean 5 m, 95% confidence interval 4 to 7) compared to daytime (mean 8 m, 95% CI 6 to 11) (Figure 5; Table S2). This model only moderately explained depth variance (*R* ^2^_*c*_ = 0.26).

All behavioural states were significant predictors of maximum dive depth (df 4, *p* < 0.01, *R* ^2^_c_ = 0.74) with a large model effect size (*ω* ^2^_*p*_ = 0.6). Feeding correlated to the deepest dive depth (model estimate mean 39 m, 95% CI 30 to 49), getting relatively shallower across exploratory foraging (mean 26 m, 95% CI 21 to 34), unknown (mean 12 m, 95% CI 9 to 18), travel (mean 7 m, 95% CI 6 to 10) and rest (mean 7 m, 95% CI 5 to 9) (Figure 5; Table S2).

### 3.3 Behavioural State Diel Trends

Proportions of time spent in behavioural states were significantly different during the day compared to at night (*χ* ^2^ = 371.73, df = 5, *p* < 0.01, 95% CI = 29.2%, 34.5%). The proportion of time spent feeding was significantly different between day and night, decreasing from 31.6% to 8.9% (*χ^2^* = 193.6, *p* < 0.01, Table S3) and rest significantly increased from 11.8% to 38.1% (*χ^2^* = 199.5, *p* < 0.01, Table S3). Exploratory foraging moderately decreased from day to night, from 18.2% to 12.3% (*χ^2^* = 33.0, *p* < 0.01, Table S3). Travelling decreased from 24.2% during the day to 21.8% at night (*χ^2^* = 7.6, *p* < 0.01, Table S3). Surface time increased from 13.7% during the day to 18.2% at night (*χ^2^* = 8.1, *p* < 0.01, Table S3). Unknown states did not differ significantly between day and night.

## 4 Discussion

In this study, locations of the tagged western North Atlantic fin whales overlapped with major shipping lanes (Figure 3), which creates a fragmented foraging habitat. Habitat fragmentation alters predator–prey dynamics, although to what degree is dependent on the species (Kareiva 1987) and risk tolerance of individuals (Rohwäder and Jeltsch 2022). The whales must cross these shipping lanes during midsummer to target habitat with high spatial and temporal density of prey (Staudinger et al. 2020), a high energetic gain that is necessary for energy stores (Brodie 1975; Christiansen et al. 2013). Body size is thought to be advantageous in fragmented habitat due to increased gap crossing ability (Hillaert et al. 2018), and these whales have exploited the most extreme size on the planet (Goldbogen et al. 2019). However, fragmentation in this shallow area is driven by traffic, in which case manoeuvrability is crucial for reducing mortality risk. Body size for these large whales may be disadvantageous since they face reduced three‐dimensional movement space which increases the likelihood of collisions with vessels, as the whales' maximum length exceeds the water depth in many areas (Lomac‐MacNair et al. 2022). These vertical movement patterns highlight the potential risk for these whales to interact with large ships.

Habitat use evolved through natural selection can become maladaptive with novel anthropogenic threats, as demonstrated by these tagged fin whales on urbanised foraging grounds. Overall, the whales spent 67.2% of their time at shallow depths (< 10 m) that put them at increased risk for collisions with vessels, as most large container ships' drafts are ca. 10 m (Garrison et al. 2022). At night, the mean maximum dive depth observed in this sample of fin whales was only 5 m, so they are at the greatest risk of ship strike at night when mariners have the greatest limitation for visual detection of whales (Gende et al. 2019). This is much shallower than the recorded nighttime 30 m median depth in the deeper Southern California Bight habitat (Keen et al. 2019). The whales also have a high risk of ship strike during the day (mean max depth 8 m) compared to 96 m in the Southern California Bight (Keen et al. 2019), though there is more variability in the distribution of dive depths than at night. These are likely conservative estimates of vertical body position in the water, as tags are placed in the middle of the ~20 m long body (NOAA 2024) and the head or tail of the whale will extend shallower than the tag depth during descent and ascent of a dive. Management strategies in the eastern United States do not currently account for these diving behaviour patterns, which can inform current models for ship strike risk. However, these may also be better informed by fine‐scale spatial overlap with oncoming ships, which is an avenue for future study.

Foraging ecology likely drives observed diel dive patterns. Activity of mobile prey can influence predator movement, particularly if prey have access to spatial refuge (Sih 1984). In particular, strong diel behaviour of prey can result in diel patterns in the foraging behaviour of predators (Romero and Harwood 2010; Bartes et al. 2025). Tagged fin whales were observed feeding only on sand lance in CATS tag video and field behavioural observations. Sand lance are a lipid rich, high value prey migrate through the water column during the day and often burrow in the sand overnight (Staudinger et al. 2020). While humpback whales ( *Megaptera novaeangliae* ) near this region have strategies for driving the prey out of the sand (Hain et al. 1995; Friedlaender et al. 2009), similar behavioural strategies have not been observed in fin whales and they may rest instead. Resting fin whales often moved in a large circle before returning to within 4–16 km of their starting point the next day possibly due to drifting during tidal movements of the water (Figure S1; Table S4). Therefore, the substrate may act as an effective spatial refuge for sand lance against fin whale predation at night. Under these circumstances, prey habitat usage and spatial distributions would drive predator foraging behaviour and vertical water column use (Sih 1984). Therefore, sand lance may be driving the observed differences in maximum depth of dives for these fin whales.

Diving behaviour in other feeding areas may also be driven by prey behaviour. Fin whales in the Southern California Bight and Azores, which feed primarily on euphausiids, also display diel dive trends (Keen et al. 2019; Fonseca et al. 2022). This behaviour may be related to how euphausiids vertically migrate to the ocean surface at night, although three‐dimensional movement was not concurrently analysed (Youngbluth 1976; Fonseca et al. 2022). Even the deepest behaviours in this study had similar mean depths (26 and 39 m) to the shallowest behaviours in the Azores (20–40 m) (Fonseca et al. 2022), demonstrating the vertical movement limitations of the ~50 m continental shelf depth (GEBCO Compilation Group 2025). Further studies on fin whale diving behaviour while consuming different prey types, and more broadly prey behaviour, are warranted.

These data must be considered in the context which they were collected. Time spent foraging during the day may be impacted by seasonality (Víkingsson 1997), so the activity budget described here represents a snapshot of behaviour during the mid‐summer in this shallow‐water habitat. The data are heavily skewed towards males, with only a single tag deployed on a female, so studies exploring whether there is a population level sex‐bias in this habitat during the mid‐summer are necessary to determine if our data are representative of the demographics for this season. Males in other mammalian taxa display greater risk‐taking behaviour than females (Jolles et al. 2015; Akinrinade et al. 2023). It is possible that males are continuing to forage in this area despite increased mortality risk from ships. However, the one tagged female in this study did not display marked differences in behavioural states compared to its paired male. More tag data from females are needed to assess potential sex differences in the activity budget and risk tolerance in this population on the foraging grounds. Additionally, analysing dive behaviours over a longer period of days to weeks could provide better insight into ship strike risk in this habitat, as our tags were limited to less than 1 day of data for each individual sampled.

This work provides critical data on the behaviours of a massive diel predator in a shallow water habitat, including some of the first details of the fine‐scale behaviour of endangered fin whales on the continental shelf in southern New England. In these habitats, some predators must cross dynamic mortality threats (i.e., heavy traffic) to reach foraging ranges with aggregations of high‐value prey, in which case site fidelity has become maladaptive in the Anthropocene (Merkle et al. 2022). Fine‐scale data such as these should be considered in risk assessment models, such as ship injury and fatality predictions, and reduced vessel speed zones.

## Author Contributions

**Dana L. Adcock:** conceptualization (supporting), data curation (lead), formal analysis (lead), investigation (supporting), methodology (equal), validation (equal), visualization (lead), writing – original draft (lead). **Douglas P. Nowacek:** conceptualization (lead), data curation (supporting), funding acquisition (lead), investigation (lead), project administration (lead), resources (equal), supervision (supporting), writing – review and editing (equal). **Susan E. Parks:** conceptualization (lead), data curation (supporting), funding acquisition (supporting), investigation (lead), methodology (equal), project administration (supporting), resources (equal), supervision (lead), validation (equal), writing – review and editing (equal).

## Funding

This material is based upon work supported by the U.S. Department of Energy's Office of Energy Efficiency and Renewable Energy (EERE) and the Bureau of Ocean Energy Management (BOEM) under the Wind Energy Technologies Office (WETO) Award Number DE‐EE0010287. Dana Adcock was supported by a Air Force Office of Scientific Research (FA9550‐23‐F‐0014).

## Ethics Statement

Fin whale tagging and biological samples were collected under the National Marine Fisheries Service Permits 27272‐01, 23644‐03 and 22156‐04 and IACUC approval from Syracuse University.

## Conflicts of Interest

The authors declare no conflicts of interest.

## Supporting information


**Figure S1:** Comparison of overnight rest and travel.
**Table S1:** Model selection. For each full model, we compared a set of nested effects models using the Akaike Information Criterion (AIC). (A) The model including State as a fixed effect and random intercepts for Individual and GroupID (AIC = 3722) had the lowest AIC value (ΔAIC > 30) compared to alternative models, indicating that these fixed and random effects contribute significantly to explaining variation in log‐transformed maximum dive depth. Additionally, the Akaike weight is 1, indicating it is likely the best model of those four. (B) The model including Period as a fixed effect and random intercepts for Individual and GroupID (AIC = 11,153) had the lowest AIC value (ΔAIC > 6) compared to alternative models, indicating that Period also contributes to explaining variation in log‐transformed maximum dive depth. Models in part B do not include twilight periods as data is split into night and day. The Akaike weight is 0.95, indicating it is likely the best model of those four.
**Table S2:** Coefficients of fixed effects in mixed models. Predictors are statistically significant in both models (*p* < 0.01). State model is lmer(log_Dive_Max ~ State + (1 | Individual) + (1 | GroupID), data = data). Period model is lmer(log_Dive_Max ~ Period + (1 | Individual) + (1 | GroupID), data = data). *Model intercept.
**Table S3:** Pairwise chi‐squared comparison of behavioural state proportions during the day versus night. The 95% CI represents the difference between day and night.
**Table S4:** Overnight displacement of tagged whales. All whales included in the table had tags with suction cups attached overnight. Starting and ending locations reflect closest GPS positions before sunset and after sunrise. Displacement measures the distance (km) between these two points. The known resting whale with high GPS sampling had a displacement of 8 km, while the travelling pair had a displacement of 46 km.

## Data Availability

Relevant code and data are available at https://doi.org/10.5061/dryad.n2z34tnd3.
