# Supplementary material for: Fin Whale (Balaenoptera physalus) Diel Dive Patterns in a Shallow Fragmented Foraging Habitat
Source: Ecol Evol. 2026 Sep 10;16(9):e74313. doi: 10.1002/ece3.74313 (PMC13561636; doi:10.1002/ece3.74313)
Supplement: Supplementary file 1 [file ECE3-16-e74313-s001.docx]

**Figure S1. Comparison of Overnight Rest and Travel**

**Table S1. Model Selection.** For each full model, we compared a set of nested effects models using the Akaike Information Criterion (AIC). A) The model including State as a fixed effect and random intercepts for Individual and GroupID (AIC = 3722) had the lowest AIC value (ΔAIC > 30) compared to alternative models, indicating that these fixed and random effects contribute significantly to explaining variation in log-transformed maximum dive depth. Additionally, the Akaike weight is 1, indicating it is likely the best model of those four. B) The model including Period as a fixed effect and random intercepts for Individual and GroupID (AIC = 11153) had the lowest AIC value (ΔAIC > 6) compared to alternative models, indicating that Period also contributes to explaining variation in log-transformed maximum dive depth. Models in part B do not include twilight periods as data is split into night and day. The Akaike weight is 0.95, indicating it is likely the best model of those four.

**A)**

| **Model (Effect Type)** | **AIC** | Δ**AIC** | **Akaike Weight** |
| --- | --- | --- | --- |
| State (Fixed) + Individual (Random) + Group (Random) | 3722 | 0 | 1 |
| State (Fixed) + Individual (Random) | 3752 | 30 | 0 |
| State (Fixed) + Group (Random) | 5754 | 2032 | 0 |
| Null | 5754 | 2032 | 0 |

**B)**

| **Model (Effect Type)** | **AIC** | Δ**AIC** | **Akaike Weight** |
| --- | --- | --- | --- |
| Period (Fixed) + Individual (Random) + Group (Random) | 11153 | 0 | 0.95 |
| Period (Fixed) + Group (Random) | 11158 | 6 | 0.05 |
| Period (Fixed) + Individual (Random) | 11178 | 25 | 0 |
| Null | 11227 | 74 | 0 |

**Table S2. Coefficients of fixed effects in mixed models.** Predictors are statistically significant in both models (p < 0.01). State model is lmer(log_Dive_Max ~ State + (1 | Individual) + (1 | GroupID), data = data). Period model is lmer(log_Dive_Max ~ Period + (1 | Individual) + (1 | GroupID), data = data). *Model intercept.

| **Model** | **Predictor** | **Beta** | **95% Confidence Interval** |
| --- | --- | --- | --- |
| State | Feeding* | 3.68 | 3.46, 3.91 |
|  | Exploratory Foraging | -0.37 | -0.45, -0.30 |
|  | Unknown | -1.09 | -1.31, -0.86 |
|  | Travel | -1.55 | -1.62, -1.48 |
|  | Rest | -1.61 | -1.68, -1.54 |
| Period | Day* | 2.22 | 1.97, 2.48 |
|  | Night | -0.37 | -0.45, -0.29 |

**Table S3. Pairwise chi-squared comparison of behavioral state proportions during the day versus night.** The 95% CI represents the difference between day and night.

| **State** | **X-squared** | **95% Confidence Interval** | **p-value** | **Adjusted p-value** |
| --- | --- | --- | --- | --- |
| Feeding | 193.6 | -16.7%, -12.8% | < 0.01 | < 0.01 |
| Exploratory Foraging | 33.0 | -6.8%, -3.4% | < 0.01 | < 0.01 |
| Traveling | 7.6 | -5.9%, -1.0% | < 0.01 | < 0.01 |
| Resting | 199.5 | 16.0%, 21.1% | < 0.01 | < 0.01 |
| Surface | 8.1 | 1.4%, 7.8% | < 0.01 | < 0.01 |
| Unknown | 0.3 | -0.3%, 0.6% | < 0.01 | < 0.01 |

**Table S4. Overnight Displacement of Tagged Whales.** All whales included in the table had tags with suction cups attached overnight. Starting and ending locations reflect closest GPS positions before sunset and after sunrise. Displacement measures the distance (km) between these two points. The known resting whale with high GPS sampling had a displacement of 8 km, while the traveling pair had a displacement of 46 km.

| **Whale ID** | **Starting Location (Latitude, Longitude)** | **Ending Location (Latitude, Longitude)** | **Displacement (km)** |
| --- | --- | --- | --- |
| Bp23_211a | 41°05'34"N 70°49'19"W | 41°06'52"N 70°47'19"W | 4 |
| Bp23_212d | 41°05'45"N, 70°46'55"W | 41°04'56"N 70°57'11"W | 14 |
| Bp23_212e & Bp23_212f (paired) | 41°04'01"N 70°49'12"W | 40°49'30"N 71°15'33"W | 46 |
| Bp23_213c | 41°05'31"N 70°45'58"W | 41°04'38"N 70°51'15"W | 8 |
| Bp24_204a | 40°52'34"N 71°47'47"W | 40°51'50"N 71°52'03"W | 6 |
| Bp24_205d | 40°52'01"N 71°54'46"W | 40°46'33"N 71°56'23"W | 10 |
| Bp24_209d | 39°52'59"N 73°08'46"W | 39°51'44"N 73°02'23"W | 9 |
